# *Allium*‐Derived Compounds Against Multidrug‐Resistant Bacteria: Antibacterial Activity and Synergistic Effects

**DOI:** 10.1002/cmdc.202501034

**Published:** 2026-04-27

**Authors:** Antonio Sorlózano‐Puerto, Yariel Figueroa‐Vega, Pablo Martín‐Valenzuela, José Manuel de la Torre‐Ramírez, Alberto Baños, José Gutiérrez‐Fernández

**Affiliations:** ^1^ Department of Microbiology Faculty of Medicine University of Granada and Instituto de Investigación Biosanitaria ibs.GRANADA Granada Spain; ^2^ DMC Research Center Alhendín Granada Spain; ^3^ Laboratory of Microbiology Hospital Universitario Virgen de las Nieves Granada Spain

**Keywords:** *allium*, antibiotic resistance, drug synergism, organosulfur compounds

## Abstract

**Background and objectives**: Antibiotic resistance is a major global health challenge, driven by misuse in medicine and agriculture and the scarcity of new drugs. *Allium*‐derived organosulfur molecules have shown antimicrobial and synergistic effects with antibiotics. This study aimed to evaluate the in vitro antibacterial activity of four *Allium*‐derived compounds and their potential synergy with clinical antibiotics against multidrug‐resistant bacteria.

**Methods**: A total of 330 multidrug‐resistant clinical isolates were analyzed, including Gram‐positive cocci and Gram‐negative bacilli. Antibacterial activity of propyl‐propane‐thiosulfinate, propyl‐propane‐thiosulfonate (PTSO), butyl‐butane‐thiosulfinate, and butyl‐butane‐thiosulfonate was determined by broth microdilution. Synergy with conventional antibiotics was assessed in 24 representative isolates using the checkerboard method, with interactions classified by the fractional inhibitory concentration index.

**Results**: PTSO exhibited the highest activity, with MIC_50_ of 4–8 µg/mL and MIC_90_ up to 8 µg/mL against *S. aureus*, *E. faecalis*, and *S. agalactiae*. Activity against Gram‐negative bacteria was limited, though PTSO was the most active. Checkerboard assays showed predominantly indifferent interactions, but partial synergy with aminoglycosides was observed in both Gram‐positive and Gram‐negative bacteria.

**Conclusion**: PTSO demonstrated the most significant activity against multidrug‐resistant Gram‐positive bacteria, with limited impact on Gram‐negative strains, and partial synergy with aminoglycosides suggests a potential role for combination therapy.

## Graphical abstract

This study evaluates four compounds derived from the genus *Allium* against 330 multidrug‐resistant bacteria. Propyl‐propane‐thiosulfonate (PTSO) exhibits the highest activity, particularly against Gram‐positive strains, while Gram‐negative bacteria are less affected. Limited synergy is observed, mainly with aminoglycosides, suggesting potential use of PTSO in combination therapies depending on bacterial type.

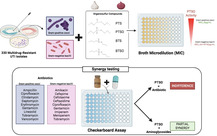

## 1 Introduction

The phenomenon of bacterial resistance to antibiotics is regarded as one of the most significant challenges currently facing clinical medicine and public health on a global scale. Since the introduction of antibiotics into medical practice, their effectiveness has resulted in a significant decrease in mortality and morbidity associated with bacterial infections. However, the inappropriate use of these drugs in clinical practice, self‐medication, their prophylactic and therapeutic use in animal production, and the lack of new antibiotics on the market have favored the emergence and spread of multi‐resistant bacteria. This has resulted in a situation in which infections that were previously treatable are now a growing risk of complications, prolonged hospital stays, increased healthcare costs, and higher morbidity and mortality rates [1, 2].

It is imperative to investigate novel therapeutic alternatives to expand the available antimicrobial arsenal and, concurrently, reestablish the efficacy of existing antibiotics. A particular area of research that has garnered significant attention in recent years pertains to the study of natural compounds that exhibit antimicrobial properties. The use of plants is valued for their low cost, few side effects, easy availability, and richness in active compounds, which is why the World Health Organization recommends assessing their therapeutic potential [3]. Species of the genus *Allium* have been widely studied due to their longstanding culinary and medicinal use, documented in ancient Sumerian, Indian, Egyptian, Greek, and Roman civilizations [4]. The secondary metabolites present in these species, particularly organosulfur compounds, such as allicin, have demonstrated significant antimicrobial properties in various studies, including the inhibition of bacterial growth, the alteration of cell structures, and interference in processes essential for the survival of pathogenic bacteria. The findings indicate that the *Allium* genus is a promising source of natural agents with potential application in antimicrobial therapy [5, 6].

Beyond the direct antibacterial activity of these compounds, it is of particular interest to analyze their possible interactions with commonly used antibiotics. The evaluation of synergistic effects between metabolites derived from *Allium* spp. and conventional antibiotics has the potential to represent an innovative strategy in the fight against resistance. Pharmacological synergy has been demonstrated to possess the capacity to enhance the efficacy of established treatments. Furthermore, it has been shown to enable the reduction of doses, to decrease associated toxicity, and, most significantly, to slow down the development of new bacterial resistance [7, 8]. Such integration has the potential to mark a substantial advancement in the quest for effective solutions to the pressing global challenge of bacterial resistance, thereby contributing to the maintenance of the efficacy of antimicrobial treatments [9].

The aim of this study was to evaluate in vitro the antibacterial activities of four organosulfur compounds that are derived from *Allium* spp., propyl‐propane‐thiosulfinate (PTS), propyl‐propane‐thiosulfonate (PTSO), butyl‐butane‐thiosulfinate (BTS), and butyl‐butane‐thiosulfonate (BTSO), against multidrug‐resistant Gram‐positive and Gram‐negative bacteria. Moreover, the study analyzed the potential synergistic effect of the most active compound in combination with commonly used clinical antibiotics.

## 2 Materials and Methods

### 2.1 Bacterial Isolates

A total of 230 Gram‐positive cocci were analyzed: 120 methicillin‐resistant *Staphylococcus aureus* (MRSA), showing combined resistance to aminoglycosides (89 isolates), fluoroquinolones (100 isolates), macrolides (78 isolates), and/or lincosamides (55 isolates); 60 *Enterococcus faecalis*, of which 53 were resistant to fluoroquinolones; and 50 *Streptococcus agalactiae*, of which three were resistant to fluoroquinolones. In addition, 120 Gram‐negative bacilli were included: 50 *Klebsiella pneumoniae*, all resistant to beta‐lactams, with combined resistance to aminoglycosides (7 isolates) and/or fluoroquinolones (41 isolates); 40 *Acinetobacter baumannii*, all resistant to beta‐lactams and fluoroquinolones, with combined resistance to aminoglycosides (37 isolates); and 30 *Pseudomonas aeruginosa*, resistant to fluoroquinolones (16 isolates), aminoglycosides (11 isolates), and/or beta‐lactams (17 isolates).

All isolates analyzed in this study were recovered between 2015 and 2024 from urine cultures processed for the diagnosis of urinary tract infections at the Microbiology Laboratory of the Virgen de las Nieves University Hospital (Granada, Spain), a reference healthcare center serving a population of ≈ 334,000 inhabitants. Urine samples were considered positive when bacterial counts reached ≥10^5^ colony‐forming units per milliliter (CFU/mL).

Species identification was performed using mass spectrometry (MALDI‐TOF, Bruker Daltonik GmbH, Bremen, Germany) or the automated MicroScan system (Beckman Coulter, Brea, California, USA), following the routine microbiology laboratory workup [10]. Antimicrobial susceptibility testing was carried out with the MicroScan system.

The isolates were preserved at −20°C and made available for the purposes of this investigation.

For quality control, the following reference strains were included in all procedures: *S. aureus* ATCC 29213, *E. faecalis* ATCC 29212, and *K. pneumoniae* ATCC 700603.

### 2.2 PTS, PTSO, BTS, BTSO, and Antibiotics

PTS, PTSO, BTS, and BTSO (97% purity) were obtained from Enzim‐Orbita Agroalimentares LDA (Tavira, Portugal) and dissolved in Tween‐80 at a final concentration of 500 g/L. In the genus *Allium*, sulfur‐derived compounds, such as BTS and PTS, originate from the corresponding S‐alkyl‐L‐cysteine sulfoxides after tissue disruption. The enzyme alliinase catalyzes the cleavage of these sulfoxides, generating unstable sulfenic acids (butyl‐ or propyl‐sulfenic acid), which spontaneously condense to form the respective thiosulfinates. Through subsequent oxidation, thiosulfinates are further converted into thiosulfonates (BTSO and PTSO), secondary metabolites with sulfur atoms in higher oxidation states [11].

All antibiotics used in the checkerboard combination assays were purchased from Sigma‐Aldrich (Madrid, Spain) and diluted according to the manufacturer's instructions.

### 2.3 Determination of the Minimum Inhibitory Concentrations (MICs)

The antibacterial activity of PTS, PTSO, BTS, and BTSO was evaluated against all 330 isolates using the broth microdilution method in cation‐adjusted Mueller–Hinton broth (CAMHB). Assays were performed in 96‐well round‐bottom microtiter plates, with bacterial suspensions adjusted to a final concentration of 1 x 10^5^ CFU/mL in each well. Each plate contained negative controls consisting of medium only and 11 serial twofold dilutions of each organosulfur compound. In a separate round‐bottom plate, positive controls consisting of a bacterial suspension without organosulfur compounds were included. The concentration ranges were as follows: PTS, 2–2048 μg/mL for all isolates; PTSO, 0.125–128 μg/mL for Gram‐positive cocci and 8–8192 μg/mL for Gram‐negative bacilli; and BTS and BTSO, 0.125–128 μg/mL for Gram‐positive cocci and 2–2048 μg/mL for Gram‐negative bacilli. Thus, the final concentration of Tween‐80 in all wells was < 1%.

The Minimum Inhibitory Concentration (MIC) was defined as the lowest concentration to completely inhibit the visible growth of a microorganism after overnight incubation. MIC_50_ and MIC_90_ values were defined as the lowest concentrations of the substance at which 50% and 90% of the isolates were inhibited, respectively. MIC values of PTS, PTSO, BTS, and BTSO were compared across bacterial groups using the Kruskal–Wallis test. When significant differences were observed, pairwise comparisons were performed with Dunn's *post hoc* test and Holm correction. Data analysis was performed using the software R (version 4.4.3). A *p* value < 0.05 was considered statistically significant.

### 2.4 Checkerboard Synergy Method

The combined antibacterial activity of substance A (the organosulfur compound with the highest activity in prior microdilution assays) and substance B (antibiotic) was evaluated using the checkerboard microdilution method in sterile 64‐well round‐bottom microtiter plates (8 × 8 format) with CAMHB as the culture medium, following previously described protocols [12, 13], a total of 24 clinical isolates (four per bacterial species) were selected from those previously characterized strains.

Each well was initially filled with 100 µL of CAMHB. Substance A was dissolved in CAMHB at four times the maximum test concentration, and 100 µL was added to column 8 (A8–H8), followed by twofold serial dilutions along each row to column 2 (A2–H2). Substance A was not added to column 1. Substance B was prepared in CAMHB at twice the target concentration range. Each concentration was prepared separately in tubes, and 100 µL was added to row A (A1–A8). The procedure was repeated for rows B–G with their corresponding concentrations. Substance B was not added to row H. Thus, row H and column 1 were reserved for testing substance A and substance B alone, respectively, while well H1 served as the growth control. This arrangement generated a two‐dimensional concentration matrix with substance A diluted across rows and substance B across columns. The final well volume was 200 µL. Detailed concentration ranges for each bacterial strain are provided in Table 1.

**TABLE 1:** Concentrations (in μg/mL) of substances A and B used in the 8 × 8 checkerboard assay.

Substance A	Bacteria	Column	1	2	3	4	5	6	7	8
PTSO	*S. aureus*, *E. faecalis* and *S. agalactiae*	Concentrations	0	0.5	1	2	4	8	16	32
	*K. pneumoniae* and *A. baumannii*		0	4	8	16	32	64	128	256
	*P. aeruginosa*		0	8	16	32	64	128	256	512

Substance B	Bacteria	Row	A	B	C	D	E	F	G	H
Ampicillin	*S. aureus*, *E. faecalis* and *S. agalactiae*	Concentrations	32	16	8	4	2	1	0.5	0
Ciprofloxacin			32	16	8	4	2	1	0.5	0
Clindamycin			32	16	8	4	2	1	0.5	0
Daptomycin			2	1	0.5	0.25	0.125	0.06	0.03	0
Erythromycin			32	16	8	4	2	1	0.5	0
Gentamicin			32	16	8	4	2	1	0.5	0
Linezolid			2	1	0.5	0.25	0.125	0.06	0.03	0
Tobramycin			32	16	8	4	2	1	0.5	0
Vancomycin			2	1	0.5	0.25	0.125	0.06	0.03	0
Amikacin	*K. pneumoniae*, *A. baumannii* and *P. aeruginosa*		32	16	8	4	2	1	0.5	0
Cefepime			32	16	8	4	2	1	0.5	0
Cefotaxime			32	16	8	4	2	1	0.5	0
Ceftazidime			32	16	8	4	2	1	0.5	0
Ciprofloxacin			32	16	8	4	2	1	0.5	0
Gentamicin			32	16	8	4	2	1	0.5	0
Imipenem			32	16	8	4	2	1	0.5	0
Meropenem			32	16	8	4	2	1	0.5	0
Tobramycin			32	16	8	4	2	1	0.5	0

Bacterial inoculum was prepared from 2 to 3 colonies of overnight cultures, suspended in 0.9% NaCl to a 0.5 McFarland standard (≈ 10^8^ CFU/mL), and subsequently diluted 1:100 in CAMHB. Twenty microliters of the suspension were added to each well, yielding a final inoculum of 1 × 10^5^ CFU/mL. Plates were incubated at 36 ± 1°C for 24 h without shaking.

All assays were performed in triplicate. MICs were defined as the lowest concentration inhibiting visible growth. MICs of individual substances (MIC_A and MIC_B) were determined in wells containing only that agent (row H and column 1, respectively), while combination MICs were recorded from wells along the growth/no‐growth boundary. When no inhibitory effect was observed at the highest concentration tested, the MIC was assigned to the next higher concentration above the tested range. The MIC value present in at least two of three replicates was reported as the final MIC [14].

For combination testing, Fractional Inhibitory Concentrations (FICs) were calculated as: FIC_A = MIC_A_comb/MIC_A and FIC_B = MIC_B_comb/MIC_B. The fractional inhibitory concentration index (FICI) was defined as FICI = FIC_A + FIC_B. Interactions were classified as synergistic (FICI < 0.5), partially synergistic (0.5 ≤ FICI < 1.0), indifferent (1 ≤ FICI < 4.0), or antagonistic (FICI ≥ 4.0) [15].

## 3 Results

### 3.1 Antibacterial Activity of Organosulfur Compounds

The antibacterial activity of PTS, PTSO, BTS, and BTSO was evaluated against six clinically relevant bacterial species, including both Gram‐positive and Gram‐negative organisms. The results, presented in Table 2, include MIC ranges as well as MIC_50_ and MIC_90_ values (in µg/mL). The MIC distributions were compared for each bacterial species using the Kruskal–Wallis test, followed by Dunn's *post hoc* test for pairwise comparisons (Table 3).

**TABLE 2:** In vitro activity of PTS, PTSO, BTS, and BTSO (in µg/mL) against isolated clinical strains.

	MICs range	MIC_50_	MIC_90_
*S. aureus*			
PTS	64–256	64	128
PTSO	1–16	8	8
BTS	8–64	16	32
BTSO	8–64	16	16
*E. faecalis*			
PTS	32–256	128	128
PTSO	0.25–8	4	8
BTS	16–64	32	32
BTSO	4–32	16	16
*S. agalactiae*			
PTS	64–128	64	128
PTSO	0.5–8	4	8
BTS	1–>128	16	64
BTSO	1–32	8	16
*K. pneumoniae*			
PTS	16–512	128	256
PTSO	16–256	128	256
BTS	32–>2048	2048	>2048
BTSO	16–>2048	512	1024
*A. baumannii*			
PTS	32–512	64	128
PTSO	≤8–256	32	64
BTS	64–>2048	128	256
BTSO	32–512	64	128
*P. aeruginosa*			
PTS	1024–>2048	1024	2048
PTSO	128–>8192	512	>8192
BTS	256–>2048	1024	>2048
BTSO	128–>2048	512	>2048

**TABLE 3:** Comparison of MIC distributions of organosulfur compounds using Kruskal–Wallis and Dunn's tests.

	*p* value (Kruskal–Wallis)	*p* value (Dunn)
*S. aureus*		
PTS/PTSO	p < 0.001	p < 0.001
PTS/BTS		p < 0.001
PTS/BTSO		p < 0.001
PTSO/BTS		p < 0.001
PTSO/BTSO		p=0.015
BTS/BTSO		p=0.489
*E. faecalis*		
PTS/PTSO	p < 0.001	p < 0.001
PTS/BTS		p < 0.001
PTS/BTSO		p < 0.001
PTSO/BTS		p < 0.001
PTSO/BTSO		p=0.011
BTS/BTSO		p < 0.001
*S. agalactiae*		
PTS/PTSO	p < 0.001	p < 0.001
PTS/BTS		p < 0.001
PTS/BTSO		p < 0.001
PTSO/BTS		p < 0.001
PTSO/BTSO		p=0.529
BTS/BTSO		p < 0.001
*K. pneumoniae*		
PTS/PTSO	p < 0.001	p=0.869
PTS/BTS		p < 0.001
PTS/BTSO		p=0.007
PTSO/BTS		p < 0.001
PTSO/BTSO		p < 0.001
BTS/BTSO		p < 0.001
*A. baumannii*		
PTS/PTSO	p < 0.014	p=0.459
PTS/BTS		p=0.415
PTS/BTSO		p=0.713
PTSO/BTS		p=0.016
PTSO/BTSO		p=0.977
BTS/BTSO		p=0.049
*P. aeruginosa*		
PTS/PTSO	p=0.112	p=0.271
PTS/BTS		p=0.952
PTS/BTSO		p=0.996
PTSO/BTS		p=0.094
PTSO/BTSO		p=0.380
BTS/BTSO		p=0.881

Among the Gram‐positive bacteria, *S. aureus* showed the highest susceptibility to PTSO, with MIC_50_ and MIC_90_ values of 8 µg/mL. The Kruskal–Wallis test revealed significant differences among the compounds (p<0.001), with Dunn's test showing statistically significant differences between most pairs (p<0.05), except for BTS vs. BTSO (p=0.489), which displayed comparable activity.

In *E. faecalis*, PTSO demonstrated the lowest MIC values (MIC_50_ = 4 µg/mL; MIC_90_ = 8 µg/mL), while BTS and BTSO exhibited moderate effectiveness. Significant differences were observed across all compounds (p<0.001), with all pairwise comparisons showing statistical significance, confirming distinct antimicrobial activity.

Similarly, *S. agalactiae* showed high susceptibility to PTSO (MIC_50_ = 4 µg/mL; MIC_90_ = 8 µg/mL) and BTSO (MIC_50_ = 8 µg/mL; MIC_90_ = 16 µg/mL). The Kruskal–Wallis test indicated significant differences among compounds (p<0.001), with all pairwise comparisons showing statistical significance except for PTSO vs. BTSO (p=0.529), highlighting their comparable antimicrobial activity in this bacterial species.

In contrast, Gram‐negative bacteria generally displayed higher MIC values, reflecting lower susceptibility to these compounds. For *K. pneumoniae*, PTS and PTSO had MIC_50_ and MIC_90_ values of 128 and 256 µg/mL, respectively. BTS and BTSO showed very limited activity, with MIC_50_ and MIC_90_ values exceeding 512 µg/mL. Although the overall Kruskal–Wallis test was significant, the comparison between PTS and PTSO was not (p=0.869). All other pairwise comparisons reached significance, pointing to clear differences in antimicrobial activity.

In *A. baumannii*, PTSO was the most active compound (MIC_50_ = 32 µg/mL; MIC_90_ = 64 µg/mL), whereas PTS and BTSO showed moderate activity, and BTS exhibited markedly higher MIC values. The Kruskal–Wallis test was significant (p=0.014), with only two pairwise comparisons (PTSO vs. BTS and BTS vs. BTSO) reaching significance, indicating clear differences between these compounds.

Lastly, *P. aeruginosa* was the most resistant species, as all compounds displayed poor activity, with MIC_50_ and MIC_90_ values ≥512 and ≥2048 µg/mL, respectively, particularly for PTSO and BTS. Neither the global test (p=0.112) nor the pairwise comparisons showed significant differences, indicating uniformly low activity across all compounds.

Overall, PTSO was the compound with the highest activity against Gram‐positive cocci and also showed some efficacy against *A. baumannii*. In contrast, against *K. pneumoniae* and *P. aeruginosa*, the activity of all compounds was limited.

### 3.2 Checkerboard Assay for PTSO in Combination With Antibiotics

The interactions between PTSO and various antibiotics were evaluated against 24 clinical isolates of Gram‐positive cocci (*S. aureus*, *E. faecalis*, and *S. agalactiae*) and Gram‐negative bacilli (*K. pneumoniae*, *A. baumannii*, and *P. aeruginosa*), as presented in Tables 4 and 5. Interactions were assessed using the FICI, a standard metric for interpreting combination effects in antimicrobial synergy testing.

**TABLE 4:** Fractional inhibitory concentration index (FICI) values obtained using the checkerboard method, and their interpretation, in the 12 clinical isolates of Gram‐positive cocci.

Bacteria	Isolate	Combination (A/B)	MIC_A	MIC_B	MIC_A_comb	MIC_B_comb	FICI	Interpretation
*S. aureus*	ID 30	PTSO/Ampicillin	32	32	16	16	1.00	Indifference
						8	0.75	Partial synergy
					32	0.5/1/2/4	1.02/1.03/1.06/1.13	Indifference
		PTSO/Ciprofloxacin		64^a^	32	0.5/1/2/4/8/16/32	1.01/1.02/1.03/1.06/1.13/1.25/1.50	Indifference
		PTSO/Clindamycin		64^a^	32	0.5/1/2/4/8/16/32	1.01/1.02/1.03/1.06/1.13/1.25/1.50	Indifference
		PTSO/Daptomycin		1	32	0.03/0.06/0.125/0.25/0.5	1.03/1.06/1.13/1.25/1.50	Indifference
		PTSO/Erythromycin		64^a^	32	0.5/1/2/4/8/16/32	1.01/1.02/1.03/1.06/1.13/1.25/1.50	Indifference
		PTSO/Gentamicin		64^a^	16	32	1.00	Indifference
						16	0.75	Partial synergy
					32	0.5/1/2/4/8	1.01/1.02/1.03/1.06/1.13	Indifference
		PTSO/Linezolid		2	16	1	1.00	Indifference
					32	0.03/0.06/0.125/0.25/0.5	1.02/1.03/1.06/1.13/1.25	Indifference
		PTSO/Tobramycin		64^a^	2	32	0.56	Partial synergy
					16	16	0.75	Partial synergy
					32	0.5/1/2/4/8	1.01/1.02/1.03/1.06/1.13	Indifference
		PTSO/Vancomycin		1	32	0.03/0.06/0.125/0.25/0.5	1.03/1.06/1.13/1.25/1.50	Indifference
	ID 80	PTSO/Ampicillin	32	32	16	16	1.00	Indifference
						8	0.75	Partial synergy
					32	0.5/1/2/4	1.02/1.03/1.06/1.13	Indifference
		PTSO/Ciprofloxacin		64^a^	32	0.5/1/2/4/8/16/32	1.01/1.02/1.03/1.06/1.13/1.25/1.50	Indifference
		PTSO/Clindamycin		64^a^	32	0.5/1/2/4/8/16/32	1.01/1.02/1.03/1.06/1.13/1.25/1.50	Indifference
		PTSO/Daptomycin		1	32	0.03/0.06/0.125/0.25/0.5	1.03/1.06/1.13/1.25/1.50	Indifference
		PTSO/Erythromycin		64^a^	32	0.5/1/2/4/8/16/32	1.01/1.02/1.03/1.06/1.13/1.25/1.50	Indifference
		PTSO/Gentamicin		64^a^	16	32	1.00	Indifference
						16	0.75	Partial synergy
					32	0.5/1/2/4/8	1.01/1.02/1.03/1.06/1.13	Indifference
		PTSO/Linezolid		2	32	0.03/0.06/0.125/0.25/0.5/1	1.02/1.03/1.06/1.13/1.25/1.50	Indifference
		PTSO/Tobramycin		64^a^	8	16/32	0.50/0.75	Partial synergy
					16	8	0.63	Partial synergy
					32	0.5/1/2/4	1.01/1.02/1.03/1.06	Indifference
		PTSO/Vancomycin		1	32	0.03/0.06/0.125/0.25/0.5	1.03/1.06/1.13/1.25/1.50	Indifference
	ID 96	PTSO/Ampicillin	32	64^a^	16	32	1.00	Indifference
					32	0.5/1/2/4/8/16	1.01/1.02/1.03/1.06/1.13/1.25	Indifference
		PTSO/Ciprofloxacin		64^a^	16	32	1.00	Indifference
					32	0.5/1/2/4/8/16	1.01/1.02/1.03/1.06/1.13/1.25	Indifference
		PTSO/Clindamycin		64^a^	32	0.5/1/2/4/8/16/32	1.01/1.02/1.03/1.06/1.13/1.25/1.50	Indifference
		PTSO/Daptomycin		1	32	0.03/0.06/0.125/0.25/0.5	1.03/1.06/1.13/1.25/1.50	Indifference
		PTSO/Erythromycin		64^a^	32	0.5/1/2/4/8/16/32	1.01/1.02/1.03/1.06/1.13/1.25/1.50	Indifference
		PTSO/Gentamicin		64^a^	16	32	1.00	Indifference
					32	0.5/1/2/4/8/16	1.01/1.02/1.03/1.06/1.13/1.25	Indifference
		PTSO/Linezolid		2	16	1	1.00	Indifference
					32	0.03/0.06/0.125/0.25/0.5	1.02/1.03/1.06/1.13/1.25	Indifference
		PTSO/Tobramycin		64^a^	8	32	0.75	Partial synergy
					16	16	0.75	Partial synergy
					32	0.5/1/2/4/8	1.01/1.02/1.03/1.06/1.13	Indifference
		PTSO/Vancomycin		1	32	0.03/0.06/0.125/0.25/0.5	1.03/1.06/1.13/1.25/1.50	Indifference
	ID 104	PTSO/Ampicillin	32	64^a^	16	32	1.00	Indifference
					32	0.5/1/2/4/8/16	1.01/1.02/1.03/1.06/1.13/1.25	Indifference
		PTSO/Ciprofloxacin		64^a^	16	32	1.00	Indifference
					32	0.5/1/2/4/8/16	1.01/1.02/1.03/1.06/1.13/1.25	Indifference
		PTSO/Clindamycin		64^a^	32	0.5/1/2/4/8/16/32	1.01/1.02/1.03/1.06/1.13/1.25/1.50	Indifference
		PTSO/Daptomycin		1	32	0.03/0.06/0.125/0.25/0.5	1.03/1.06/1.13/1.25/1.50	Indifference
		PTSO/Erythromycin		64^a^	32	0.5/1/2/4/8/16/32	1.01/1.02/1.03/1.06/1.13/1.25/1.50	Indifference
		PTSO/Gentamicin		64^a^	16	32	1.00	Indifference
						16	0.75	Partial synergy
					32	0.5/1/2/4/8	1.01/1.02/1.03/1.06/1.13	Indifference
		PTSO/Linezolid		2	16	1	1.00	Indifference
					32	0.03/0.06/0.125/0.25/0.5	1.02/1.03/1.06/1.13/1.25	Indifference
		PTSO/Tobramycin		64^a^	8	32	0.75	Partial synergy
					16	16	0.75	Partial synergy
					32	0.5/1/2/4/8	1.01/1.02/1.03/1.06/1.13	Indifference
		PTSO/Vancomycin		1	32	0.03/0.06/0.125/0.25/0.5	1.03/1.06/1.13/1.25/1.50	Indifference
*E. faecalis*	ID 392	PTSO/Ampicillin	32	8	32	0.5/1/2/4	1.06/1.13/1.25/1.50	Indifference
		PTSO/Ciprofloxacin		64^a^	16	32	1.00	Indifference
					32	0.5/1/2/4/8/16	1.01/1.02/1.03/1.06/1.13/1.25	Indifference
		PTSO/Clindamycin		64^a^	32	0.5/1/2/4/8/16/32	1.01/1.02/1.03/1.06/1.13/1.25/1.50	Indifference
		PTSO/Daptomycin		2	32	0.03/0.06/0.125/0.25/0.5/1	1.02/1.03/1.06/1.13/1.25/1.50	Indifference
		PTSO/Erythromycin		64^a^	32	0.5/1/2/4/8/16/32	1.01/1.02/1.03/1.06/1.13/1.25/1.50	Indifference
		PTSO/Gentamicin		16	16	8	1.00	Indifference
						2/4	0.63/0.75	Partial synergy
					32	0.5/1	1.03/1.06	Indifference
		PTSO/Linezolid		2	16	1	1.00	Indifference
					32	0.03/0.06/0.125/0.25/0.5	1.02/1.03/1.06/1.13/1.25	Indifference
		PTSO/Tobramycin		64^a^	16	32	1.00	Indifference
						16	0.75	Partial synergy
					32	0.5/1/2/4/8	1.01/1.02/1.03/1.06/1.13	Indifference
		PTSO/Vancomycin		1	16	0.5	1.00	Indifference
					32	0.03/0.06/0.125/0.25	1.03/1.06/1.13/1.25	Indifference
	ID 407	PTSO/Ampicillin	32	4	16	2	1.00	Indifference
					32	0.5/1	1.13/1.25	Indifference
		PTSO/Ciprofloxacin		64^a^	32	0.5/1/2/4/8/16/32	1.01/1.02/1.03/1.06/1.13/1.25/1.50	Indifference
		PTSO/Clindamycin		64^a^	32	0.5/1/2/4/8/16/32	1.01/1.02/1.03/1.06/1.13/1.25/1.50	Indifference
		PTSO/Daptomycin		2	16	1	1.00	Indifference
						0.5	0.75	Partial synergy
					32	0.03/0.06/0.125/0.25	1.02/1.03/1.06/1.13	Indifference
		PTSO/Erythromycin		64^a^	32	0.5/1/2/4/8/16/32	1.01/1.02/1.03/1.06/1.13/1.25/1.50	Indifference
		PTSO/Gentamicin		64^a^	32	0.5/1/2/4/8/16/32	1.01/1.02/1.03/1.06/1.13/1.25/1.50	Indifference
		PTSO/Linezolid		2	32	0.03/0.06/0.125/0.25/0.5/1	1.02/1.03/1.06/1.13/1.25/1.50	Indifference
		PTSO/Tobramycin		64^a^	32	0.5/1/2/4/8/16/32	1.01/1.02/1.03/1.06/1.13/1.25/1.50	Indifference
		PTSO/Vancomycin		1	32	0.03/0.06/0.125/0.25/0.5	1.03/1.06/1.13/1.25/1.50	Indifference
	ID 421	PTSO/Ampicillin	32	1	32	0.5	1.50	Indifference
		PTSO/Ciprofloxacin		64^a^	32	0.5/1/2/4/8/16/32	1.01/1.02/1.03/1.06/1.13/1.25/1.50	Indifference
		PTSO/Clindamycin		64^a^	32	0.5/1/2/4/8/16/32	1.01/1.02/1.03/1.06/1.13/1.25/1.50	Indifference
		PTSO/Daptomycin		1	32	0.03/0.06/0.125/0.25/0.5	1.03/1.06/1.13/1.25/1.50	Indifference
		PTSO/Erythromycin		64^a^	32	0.5/1/2/4/8/16/32	1.01/1.02/1.03/1.06/1.13/1.25/1.50	Indifference
		PTSO/Gentamicin		64^a^	32	0.5/1/2/4/8/16/32	1.01/1.02/1.03/1.06/1.13/1.25/1.50	Indifference
		PTSO/Linezolid		2	32	0.03/0.06/0.125/0.25/0.5/1	1.02/1.03/1.06/1.13/1.25/1.50	Indifference
		PTSO/Tobramycin		64^a^	16	32	1.00	Indifference
					32	0.5/1/2/4/8/16	1.01/1.02/1.03/1.06/1.13/1.25	Indifference
		PTSO/Vancomycin		1	32	0.03/0.06/0.125/0.25/0.5	1.03/1.06/1.13/1.25/1.50	Indifference
	ID 438	PTSO/Ampicillin	32	4	16	2	1.00	Indifference
					32	0.5/1	1.13/1.25	Indifference
		PTSO/Ciprofloxacin		≤0.5	ND	ND	ND	ND
		PTSO/Clindamycin		64^a^	32	0.5/1/2/4/8/16/32	1.01/1.02/1.03/1.06/1.13/1.25/1.50	Indifference
		PTSO/Daptomycin		1	32	0.03/0.06/0.125/0.25/0.5	1.03/1.06/1.13/1.25/1.50	Indifference
		PTSO/Erythromycin		64^a^	32	0.5/1/2/4/8/16/32	1.01/1.02/1.03/1.06/1.13/1.25/1.50	Indifference
		PTSO/Gentamicin		16	16	8	1.00	Indifference
						1/2/4	0.56/0.63/0.75	Partial synergy
					32	0.5	1.03	Indifference
		PTSO/Linezolid		2	16	1	1.00	Indifference
					32	0.03/0.06/0.125/0.25/0.5	1.02/1.03/1.06/1.13/1.25	Indifference
		PTSO/Tobramycin		16	4	8	0.63	Partial synergy
					8	4	0.50	Partial synergy
					16	0.5/1/2	0.53/0.56/0.63	Partial synergy
		PTSO/Vancomycin		2	16	1	1.00	Indifference
					32	0.03/0.06/0.125/0.25/0.5	1.02/1.03/1.06/1.13/1.25	Indifference
*S. agalactiae*	ID 9	PTSO/Ampicillin	16	≤0.5	ND	ND	ND	ND
		PTSO/Ciprofloxacin		≤0.5	ND	ND	ND	ND
		PTSO/Clindamycin		≤0.5	ND	ND	ND	ND
		PTSO/Daptomycin		0.5	8	0.25	1.00	Indifference
						0.125	0.75	Partial synergy
					16	0.03/0.06	1.06/1.13	Indifference
		PTSO/Erythromycin		≤0.5	ND	ND	ND	ND
		PTSO/Gentamicin		4	8	2	1.00	Indifference
					16	0.5/1	1.13/1.25	Indifference
		PTSO/Linezolid		2	16	0.03/0.06/0.125/0.25/0.5/1	1.02/1.03/1.06/1.13/1.25/1.50	Indifference
		PTSO/Tobramycin		4	16	0.5/1/2	1.13/1.25/1.50	Indifference
		PTSO/Vancomycin		0.25	16	0.03/0.06/0.125	1.13/1.25/1.50	Indifference
	ID 133	PTSO/Ampicillin	16	≤0.5	ND	ND	ND	ND
		PTSO/Ciprofloxacin		≤0.5	ND	ND	ND	ND
		PTSO/Clindamycin		≤0.5	ND	ND	ND	ND
		PTSO/Daptomycin		0.5	4	0.125/0.25	0.50/0.75	Partial synergy
					16	0.03/0.06	1.06/1.13	Indifference
		PTSO/Erythromycin		≤0.5	ND	ND	ND	ND
		PTSO/Gentamicin		2	16	0.5/1	1.25/1.50	Indifference
		PTSO/Linezolid		1	16	0.03/0.06/0.125/0.25/0.5	1.03/1.06/1.13/1.25/1.50	Indifference
		PTSO/Tobramycin		2	16	0.5/1	1.25/1.50	Indifference
		PTSO/Vancomycin		0.5	4	0.25	0.75	Partial synergy
					16	0.03/0.06/0.125	1.06/1.13/1.25	Indifference
	ID 520	PTSO/Ampicillin	16	≤0.5	ND	ND	ND	ND
		PTSO/Ciprofloxacin		≤0.5	ND	ND	ND	ND
		PTSO/Clindamycin		≤0.5	ND	ND	ND	ND
		PTSO/Daptomycin		1	4	0.5	0.75	Partial synergy
					16	0.03/0.06/0.125/0.25	1.03/1.06/1.13/1.25	Indifference
		PTSO/Erythromycin		≤0.5	ND	ND	ND	ND
		PTSO/Gentamicin		4	16	0.5/1/2	1.13/1.25/1.50	Indifference
		PTSO/Linezolid		1	16	0.03/0.06/0.125/0.25/0.5	1.03/1.06/1.13/1.25/1.50	Indifference
		PTSO/Tobramycin		4	16	0.5/1/2	1.13/1.25/1.50	Indifference
		PTSO/Vancomycin		0.5	16	0.03/0.06/0.125/0.25	1.06/1.13/1.25/1.50	Indifference
	ID 521	PTSO/Ampicillin	16	≤0.5	ND	ND	ND	ND
		PTSO/Ciprofloxacin		≤0.5	ND	ND	ND	ND
		PTSO/Clindamycin		≤0.5	ND	ND	ND	ND
		PTSO/Daptomycin		1	4	0.5	0.75	Partial synergy
					8	0.125/0.25	0.63/0.75	Partial synergy
					16	0.03/0.06	1.03/1.06	Indifference
		PTSO/Erythromycin		≤0.5	ND	ND	ND	ND
		PTSO/Gentamicin		8	8	4	1.00	Indifference
					16	0.5/1/2	1.06/1.13/1.25	Indifference
		PTSO/Linezolid		1	16	0.03/0.06/0.125/0.25/0.5	1.03/1.06/1.13/1.25/1.50	Indifference
		PTSO/Tobramycin		8	8	4	1.00	Indifference
					16	0.5/1/2	1.06/1.13/1.25	Indifference
		PTSO/Vancomycin		0.5	8	0.25	1.00	Indifference
					16	0.03/0.06/0.125	1.06/1.13/1.25	Indifference

ND: The MIC value of the antibiotic (MIC_B) was lower than the lowest concentration tested; therefore, FICI calculation could not be performed.

a For FICI determination, absence of inhibition at the maximum concentration tested (32 μg/mL) led to assigning the MIC as the next higher value, 64 μg/mL.

**TABLE 5:** FICI values obtained using the chequerboard method, and their interpretation, in the 12 clinical isolates of Gram‐negative bacilli.

Bacteria	Isolate	Combination (A/B)	MIC_A	MIC_B	MIC_A_comb	MIC_B_comb	FICI	Interpretation
*K. pneumoniae*	ID 903	PTSO/Amikacin	128	64^a^	128	0.5/1/2/4/8/16/32	1.01/1.02/1.03/1.06/1.13/1.25/1.50	Indifference
		PTSO/Cefepime		64^a^	128	0.5/1/2/4/8/16/32	1.01/1.02/1.03/1.06/1.13/1.25/1.50	Indifference
		PTSO/Cefotaxime		64^a^	128	0.5/1/2/4/8/16/32	1.01/1.02/1.03/1.06/1.13/1.25/1.50	Indifference
		PTSO/Ceftazidime		64^a^	128	0.5/1/2/4/8/16/32	1.01/1.02/1.03/1.06/1.13/1.25/1.50	Indifference
		PTSO/Ciprofloxacin		64^a^	64	32	1.00	Indifference
					128	0.5/1/2/4/8/16	1.01/1.02/1.03/1.06/1.13/1.25	Indifference
		PTSO/Gentamicin		64^a^	128	0.5/1/2/4/8/16/32	1.01/1.02/1.03/1.06/1.13/1.25/1.50	Indifference
		PTSO/Imipenem		64^a^	128	0.5/1/2/4/8/16/32	1.01/1.02/1.03/1.06/1.13/1.25/1.50	Indifference
		PTSO/Meropenem		64^a^	128	0.5/1/2/4/8/16/32	1.01/1.02/1.03/1.06/1.13/1.25/1.50	Indifference
		PTSO/Tobramycin		64^a^	16	32	0.63	Partial synergy
					32	16	0.50	Partial synergy
					64	8	0.63	Partial synergy
					128	0.5/1/2/4	1.01/1.02/1.03/1.06	Indifference
	ID 915, ID 916, ID 920	PTSO/Amikacin	32	64^a^	32	0.5/1/2/4/8/16/32	1.01/1.02/1.03/1.06/1.13/1.25/1.50	Indifference
		PTSO/Cefepime		64^a^	32	0.5/1/2/4/8/16/32	1.01/1.02/1.03/1.06/1.13/1.25/1.50	Indifference
		PTSO/Cefotaxime		64^a^	32	0.5/1/2/4/8/16/32	1.01/1.02/1.03/1.06/1.13/1.25/1.50	Indifference
		PTSO/Ceftazidime		64^a^	32	0.5/1/2/4/8/16/32	1.01/1.02/1.03/1.06/1.13/1.25/1.50	Indifference
		PTSO/Ciprofloxacin		64^a^	32	0.5/1/2/4/8/16/32	1.01/1.02/1.03/1.06/1.13/1.25/1.50	Indifference
		PTSO/Gentamicin		64^a^	32	0.5/1/2/4/8/16/32	1.01/1.02/1.03/1.06/1.13/1.25/1.50	Indifference
		PTSO/Imipenem		64^a^	32	0.5/1/2/4/8/16/32	1.01/1.02/1.03/1.06/1.13/1.25/1.50	Indifference
		PTSO/Meropenem		64^a^	32	0.5/1/2/4/8/16/32	1.01/1.02/1.03/1.06/1.13/1.25/1.50	Indifference
		PTSO/Tobramycin		64^a^	32	0.5/1/2/4/8/16/32	1.01/1.02/1.03/1.06/1.13/1.25/1.50	Indifference
*A. baumannii*	ID 760	PTSO/Amikacin	32	64^a^	16	32	1.00	Indifference
					32	0.5/1/2/4/8/16	1.01/1.02/1.03/1.06/1.13/1.25	Indifference
		PTSO/Cefepime		64^a^	32	0.5/1/2/4/8/16/32	1.01/1.02/1.03/1.06/1.13/1.25/1.50	Indifference
		PTSO/Cefotaxime		64^a^	32	0.5/1/2/4/8/16/32	1.01/1.02/1.03/1.06/1.13/1.25/1.50	Indifference
		PTSO/Ceftazidime		64^a^	32	0.5/1/2/4/8/16/32	1.01/1.02/1.03/1.06/1.13/1.25/1.50	Indifference
		PTSO/Ciprofloxacin		64^a^	32	0.5/1/2/4/8/16/32	1.01/1.02/1.03/1.06/1.13/1.25/1.50	Indifference
		PTSO/Gentamicin		4	8	2	0.75	Partial synergy
					16	1	0.75	Partial synergy
					32	0.5	1.13	Indifference
		PTSO/Imipenem		64^a^	32	0.5/1/2/4/8/16/32	1.01/1.02/1.03/1.06/1.13/1.25/1.50	Indifference
		PTSO/Meropenem		64^a^	32	0.5/1/2/4/8/16/32	1.01/1.02/1.03/1.06/1.13/1.25/1.50	Indifference
		PTSO/Tobramycin		1	4	0.5	0.63	Partial synergy
	ID 762	PTSO/Amikacin	128	4	16	2	0.63	Partial synergy
					32	1	0.50	Partial synergy
					128	0.5	1.13	Indifference
		PTSO/Cefepime		64^a^	32	0.5/1/2/4/8/16/32	1.01/1.02/1.03/1.06/1.13/1.25/1.50	Indifference
		PTSO/Cefotaxime		64^a^	32	0.5/1/2/4/8/16/32	1.01/1.02/1.03/1.06/1.13/1.25/1.50	Indifference
		PTSO/Ceftazidime		64^a^	32	0.5/1/2/4/8/16/32	1.01/1.02/1.03/1.06/1.13/1.25/1.50	Indifference
		PTSO/Ciprofloxacin		8	128	0.5/1/2/4	1.06/1.13/1.25/1.50	Indifference
		PTSO/Gentamicin		32	64	16	1.00	Indifference
					128	0.5/1/2/4/8	1.02/1.03/1.06/1.13/1.25	Indifference
		PTSO/Imipenem		64^a^	32	0.5/1/2/4/8/16/32	1.01/1.02/1.03/1.06/1.13/1.25/1.50	Indifference
		PTSO/Meropenem		64^a^	32	0.5/1/2/4/8/16/32	1.01/1.02/1.03/1.06/1.13/1.25/1.50	Indifference
		PTSO/Tobramycin		16	16	8	0.63	Partial synergy
					32	4	0.50	Partial synergy
					64	0.5/1/2	0.53/0.53/0.63	Partial synergy
	ID 781, ID 783	PTSO/Amikacin	32	64^a^	32	0.5/1/2/4/8/16/32	1.01/1.02/1.03/1.06/1.13/1.25/1.50	Indifference
		PTSO/Cefepime		64^a^	32	0.5/1/2/4/8/16/32	1.01/1.02/1.03/1.06/1.13/1.25/1.50	Indifference
		PTSO/Cefotaxime		64^a^	32	0.5/1/2/4/8/16/32	1.01/1.02/1.03/1.06/1.13/1.25/1.50	Indifference
		PTSO/Ceftazidime		64^a^	32	0.5/1/2/4/8/16/32	1.01/1.02/1.03/1.06/1.13/1.25/1.50	Indifference
		PTSO/Ciprofloxacin		64^a^	32	0.5/1/2/4/8/16/32	1.01/1.02/1.03/1.06/1.13/1.25/1.50	Indifference
		PTSO/Gentamicin		64^a^	32	0.5/1/2/4/8/16/32	1.01/1.02/1.03/1.06/1.13/1.25/1.50	Indifference
		PTSO/Imipenem		64^a^	32	0.5/1/2/4/8/16/32	1.01/1.02/1.03/1.06/1.13/1.25/1.50	Indifference
		PTSO/Meropenem		64^a^	32	0.5/1/2/4/8/16/32	1.01/1.02/1.03/1.06/1.13/1.25/1.50	Indifference
		PTSO/Tobramycin		64^a^	32	0.5/1/2/4/8/16/32	1.01/1.02/1.03/1.06/1.13/1.25/1.50	Indifference
*P. aeruginosa*	ID 819	PTSO/Amikacin	512	8	128	4	0.75	Partial synergy
					512	0.5/1/2	1.06/1.13/1.25	Indifference
		PTSO/Cefepime		32	512	0.5/1/2/4/8/16	1.02/1.03/1.06/1.13/1.25/1.50	Indifference
		PTSO/Cefotaxime		64^a^	512	0.5/1/2/4/8/16/32	1.01/1.02/1.03/1.06/1.13/1.25/1.50	Indifference
		PTSO/Ceftazidime		32	512	0.5/1/2/4/8/16	1.02/1.03/1.06/1.13/1.25/1.50	Indifference
		PTSO/Ciprofloxacin		16	512	0.5/1/2/4/8	1.03/1.06/1.13/1.25/1.50	
		PTSO/Gentamicin		4	256	2	1.00	Indifference
					512	0.5/1	1.13/1.25	Indifference
		PTSO/Imipenem		64^a^	256	32	1.00	Indifference
					512	0.5/1/2/4/8/16	1.01/1.02/1.03/1.06/1.13/1.25	Indifference
		PTSO/Meropenem		64^a^	256	32	1.00	Indifference
					512	0.5/1/2/4/8/16	1.01/1.02/1.03/1.06/1.13/1.25	Indifference
		PTSO/Tobramycin		1	128	0.5	0.75	Partial synergy
	ID 885	PTSO/Amikacin	256	4	128	2	1.00	Indifference
					256	0.5/1	1.13/1.25	Indifference
		PTSO/Cefepime		32	256	0.5/1/2/4/8/16	1.02/1.03/1.06/1.13/1.25/1.50	Indifference
		PTSO/Cefotaxime		64^a^	256	0.5/1/2/4/8/16/32	1.01/1.02/1.03/1.06/1.13/1.25/1.50	Indifference
		PTSO/Ceftazidime		16	256	0.5/1/2/4/8	1.03/1.06/1.13/1.25/1.50	Indifference
		PTSO/Ciprofloxacin		1	256	0.5	1.50	Indifference
		PTSO/Gentamicin		2	64	1	0.75	Partial synergy
					128	0.5	0.75	Partial synergy
		PTSO/Imipenem		64^a^	256	0.5/1/2/4/8/16/32	1.01/1.02/1.03/1.06/1.13/1.25/1.50	Indifference
		PTSO/Meropenem		64^a^	256	0.5/1/2/4/8/16/32	1.01/1.02/1.03/1.06/1.13/1.25/1.50	Indifference
		PTSO/Tobramycin		≤0.5	ND	ND	ND	ND
	ID 896	PTSO/Amikacin	128	8	128	0.5/1/2/4	1.06/1.13/1.25/1.50	Indifference
		PTSO/Cefepime		64^a^	128	0.5/1/2/4/8/16/32	1.01/1.02/1.03/1.06/1.13/1.25/1.50	Indifference
		PTSO/Cefotaxime		64^a^	128	0.5/1/2/4/8/16/32	1.01/1.02/1.03/1.06/1.13/1.25/1.50	Indifference
		PTSO/Ceftazidime		64^a^	128	0.5/1/2/4/8/16/32	1.01/1.02/1.03/1.06/1.13/1.25/1.50	Indifference
		PTSO/Ciprofloxacin		≤0.5	ND	ND	ND	ND
		PTSO/Gentamicin		64^a^	128	0.5/1/2/4/8/16/32	1.01/1.02/1.03/1.06/1.13/1.25/1.50	Indifference
		PTSO/Imipenem		64^a^	128	0.5/1/2/4/8/16/32	1.01/1.02/1.03/1.06/1.13/1.25/1.50	Indifference
		PTSO/Meropenem		64^a^	128	0.5/1/2/4/8/16/32	1.01/1.02/1.03/1.06/1.13/1.25/1.50	Indifference
		PTSO/Tobramycin		64^a^	128	0.5/1/2/4/8/16/32	1.01/1.02/1.03/1.06/1.13/1.25/1.50	Indifference
	ID 902	PTSO/Amikacin	256	64^a^	256	0.5/1/2/4/8/16/32	1.01/1.02/1.03/1.06/1.13/1.25/1.50	Indifference
		PTSO/Cefepime		64^a^	256	0.5/1/2/4/8/16/32	1.01/1.02/1.03/1.06/1.13/1.25/1.50	Indifference
		PTSO/Cefotaxime		64^a^	256	0.5/1/2/4/8/16/32	1.01/1.02/1.03/1.06/1.13/1.25/1.50	Indifference
		PTSO/Ceftazidime		64^a^	256	0.5/1/2/4/8/16/32	1.01/1.02/1.03/1.06/1.13/1.25/1.50	Indifference
		PTSO/Ciprofloxacin		≤0.5	ND	ND	ND	ND
		PTSO/Gentamicin		64^a^	128	32	1.00	Indifference
					128	16	0.75	Partial synergy
					256	0.5/1/2/4/8	1.01/1.02/1.03/1.06/1.13	Indifference
		PTSO/Imipenem		64^a^	256	0.5/1/2/4/8/16/32	1.01/1.02/1.03/1.06/1.13/1.25/1.50	Indifference
		PTSO/Meropenem		64^a^	128	32	1.00	Indifference
					256	0.5/1/2/4/8/16	1.01/1.02/1.03/1.06/1.13/1.25	Indifference
		PTSO/Tobramycin		64^a^	128	32	1.00	Indifference
					256	0.5/1/2/4/8/16	1.01/1.02/1.03/1.06/1.13/1.25	Indifference

ND: The MIC value of the antibiotic (MIC_B) was lower than the lowest concentration tested; therefore, FICI calculation could not be performed.

a For FICI determination, absence of inhibition at the maximum concentration tested (32 μg/mL) led to assigning the MIC as the next higher value, 64 μg/mL.

In Gram‐positive cocci, combinations of PTSO with ciprofloxacin, clindamycin, erythromycin, and linezolid, as well as combinations with beta‐lactams and ciprofloxacin in Gram‐negative bacilli, consistently yielded FICI values above 1.0, indicating indifferent interactions. This suggests that PTSO generally does not significantly enhance or diminish the activity of these antibiotics.

Notably, a few combinations demonstrated partial synergistic effects. In Gram‐positive cocci, PTSO combined with gentamicin, tobramycin, or daptomycin yielded FICI values ranging from 0.50 to 0.75, suggesting potential enhancement of antimicrobial activity. Similarly, in Gram‐negative isolates, combinations of PTSO with gentamicin, tobramycin, or amikacin resulted in FICI values indicative of partial synergy. Among the tested antibiotics, aminoglycosides (particularly tobramycin and gentamicin) were the most frequently associated with partial synergistic interactions.

## 4 Discussion

The present study provides an evaluation of the antibacterial activity of four organosulfur compounds (PTS, PTSO, BTS, and BTSO) against clinically relevant Gram‐positive and Gram‐negative bacterial species. Among these compounds, PTSO demonstrated the strongest antibacterial activity, particularly against Gram‐positive cocci, including *S. aureus*, *E. faecalis*, and *S. agalactiae*. This is supported by the significantly low MIC_50_ and MIC_90_ values for PTSO compared with PTS, BTS, and BTSO, which highlight its efficacy against these microorganisms. These results are consistent with previous studies reporting the antibacterial properties of organosulfur compounds derived from *Allium* spp., including activity against MRSA and vancomycin‐resistant enterococci (VRE) [16, 17].

In contrast, Gram‐negative bacteria, such as *K. pneumoniae* and *P. aeruginosa,* exhibited markedly higher MIC values, indicating limited susceptibility to all the organosulfur compounds tested. This reduced activity is consistent with previous reports [17, 18] and is generally attributed to the presence of lipopolysaccharides in the outer membrane, which hinders the penetration of these molecules [19]. Nevertheless, an exception was observed with *A. baumannii*, which showed moderate susceptibility to PTSO, possibly due to variations in membrane permeability or composition that allow partial access of the compound to intracellular targets [18].

Overall, the antibacterial activity of PTSO can be attributed to its chemical stability and the reactivity of its sulfur moieties. Similar to other organosulfur compounds such as allicin, these properties promote interactions with essential bacterial enzymes and cell wall components. Its ability to disrupt key thiol‐containing enzymes and to compromise membrane integrity likely underpins its effectiveness [20, 21].

Checkerboard assays showed that PTSO usually displayed indifferent interactions with antibiotics. Indifference means that combining PTSO with antibiotics neither enhances nor reduces activity, indicating limited therapeutic benefit. In Gram‐positive cocci, combinations with ciprofloxacin, clindamycin, erythromycin, and linezolid, and in Gram‐negative bacilli with beta‐lactams and ciprofloxacin, consistently remained indifferent. However, PTSO exhibited partial synergy with aminoglycosides (gentamicin, tobramycin, and amikacin) in both Gram‐positive and Gram‐negative isolates, suggesting its potential role as an adjunct in targeted therapies, particularly against resistant strains. Partial synergy denotes a greater inhibitory effect than expected from individual activities, but not to the extent of full synergy. This observation corroborates several studies demonstrating synergistic effects of other organosulfur compounds, such as allicin, with conventional antibiotics [7, 8]. For instance, the combinations of allicin with gentamicin resulted in significant reductions in bacterial viability [9]. In contrast, most combinations with beta‐lactams, fluoroquinolones, or macrolides were indifferent, indicating that the synergistic potential of organosulfur compounds is highly dependent on the specific mechanism of action of the antibiotics involved [22].

Organosulfur compounds exert antibacterial activity through multiple pathways. They target sulfhydryl‐containing enzymes critical for bacterial metabolism and disrupt membrane integrity, thereby enhancing antibiotic uptake, particularly of aminoglycosides that require intracellular accumulation to exert bactericidal effects [16, 23]. Allicin, specifically, has been reported to enhance the action of antibiotics by altering the integrity and structure of microbial cells through the reduction of lipid content, thereby facilitating the entry of antibiotic molecules [9]. This dual mode of action may account for the low MICs observed against Gram‐positive bacteria and the partial synergistic interactions with aminoglycosides.

The observed synergy is concentration‐dependent, a phenomenon well‐documented in antimicrobial pharmacology. Antibiotic combinations may exhibit synergy only within specific concentration ranges, while subinhibitory or excessively high concentrations may result in indifferent or antagonistic interactions. This concept is particularly relevant for PTSO combinations, as partial synergy with aminoglycosides was only evident at specific concentrations in our study. Optimizing dosing strategies to maximize these concentration‐dependent synergistic effects is crucial for potential clinical application [7].

These findings suggest potential clinical interest in PTSO as a possible adjunctive candidate. Its in vitro activity against multidrug‐resistant Gram‐positive cocci and its observed partial synergy with aminoglycosides raise the possibility that PTSO could enable reduced doses of these antibiotics in future in vivo studies. Such an approach may contribute to minimizing toxicity and slowing resistance development [5], though this remains speculative pending animal model validation. The relatively limited activity against Gram‐negative organisms might be exploited to target specific infections while preserving protective microbiota, though this requires further investigation. These results suggest promise in healthcare settings where multidrug‐resistant Gram‐positive pathogens, such as MRSA and VRE, are prevalent [24], but clinical applicability awaits mechanistic understanding and in vivo efficacy data.

Despite promising results, several limitations must be acknowledged. First, this study was conducted entirely in vitro, which may not fully reflect in vivo efficacy due to differences in pharmacokinetics, bioavailability, and host‐related factors. Pharmacokinetic factors (absorption, distribution, metabolism, and excretion), bioavailability variations, host immune responses, and systemic toxicity cannot be inferred from these data and are essential for any future therapeutic development. Second, the study included a limited number of clinical isolates, potentially limiting generalizability. Third, this study lacks de novo mechanistic investigations; no proteomic, transcriptomic, membrane biophysics, or target identification experiments were performed. The mechanistic interpretations offered here are therefore inferential, based on integration with published literature on allicin and related organosulfur compounds. Definitive elucidation of the molecular targets and pathways underpinning the observed antibacterial activity and aminoglycoside synergy requires dedicated mechanistic studies. Fourth, safety and cytotoxicity profiles of PTSO and related compounds were not assessed, which is critical for clinical translation. Finally, the observation of partial synergy is concentration‐dependent and has been demonstrated only at specific concentration ranges in vitro; optimal dosing strategies for potential in vivo or clinical use cannot be determined from this dataset alone. For these reasons, the present study should be regarded as a comprehensive preclinical screening and combination mapping effort that establishes essential baseline data (spectrum, potency, and checkerboard interaction patterns).

Future research should focus on validating these findings in vivo, assessing both efficacy and toxicity in animal models, and conducting mechanistic studies to elucidate molecular targets. Formulation strategies, such as encapsulation or nanoemulsion delivery, could enhance stability and bioavailability, overcoming challenges associated with volatile organosulfur compounds [19, 25, 26]. Systematic evaluation of combination therapies, guided by concentration‐dependent synergy data, will be essential to identify optimal dosing regimens for clinical use [22].

In conclusion, PTSO emerges as a promising lead compound exhibiting robust in vitro activity against multidrug‐resistant Gram‐positive pathogens. The partial synergistic effects documented with aminoglycosides are hypothesis‐generating, suggesting potential avenues for future in vivo dose‐reduction strategies. This comprehensive preclinical dataset—defining antimicrobial spectrum, potency distributions (MIC_50_, MIC_90_), and quantitative synergy interactions (FICI)—provides an essential foundation on which mechanistic investigations and in vivo validation studies can be built. Integration of organosulfur compounds into future combination therapy strategies requires additional research to establish safety profiles, mechanistic understanding, and in vivo efficacy before any clinical translation can be considered.

## Author Contributions

**Antonio Sorlózano‐Puerto**: conceptualization, data curation, formal analysis, supervision, validation and writing – original draft. **Yariel Figueroa‐Vega**: investigations, methodology and writing – original draft. **Pablo Martín‐Valenzuela**: investigations, methodology and writing – original draft. **José Manuel de la Torre‐Ramírez**: methodology and writing – review and editing. **Alberto Baños**: conceptualization, validation and writing – review and editing. **José Gutiérrez‐Fernández**: conceptualization, formal analysis, supervision, validation and writing – review and editing.

## Funding

This research did not receive any specific grant from funding agencies in the public, commercial, or not‐for‐profit sectors. Funding for open access charge: University of Granada / CBUA.

## Ethics Statement

The study protocol was conducted in accordance with the principles of the Declaration of Helsinki. This was a noninterventional study, and no procedures involving human participants beyond routine clinical practice were performed. No additional sampling was carried out, and routine clinical protocols were not altered. Authorization to access and use the bacterial isolates was granted by the Microbiology Laboratory of the Virgen de las Nieves University Hospital (Granada, Spain).

## Conflicts of Interest

The authors declare no conflicts of interest.

## Data Availability

All data generated or analyzed during this study are included in this published article.
